# Primary Empty Sella Presenting With Anterior Hypopituitarism: Reframing a Benign Radiologic Finding Through Its Endocrine Consequences

**DOI:** 10.1016/j.aed.2025.08.013

**Published:** 2025-08-28

**Authors:** Emmeline Monique T. Ngo

**Affiliations:** Section of Endocrinology, Diabetes, and Metabolism - Department of Medicine, University of Missouri-Kansas City, Kansas City, Missouri

**Keywords:** empty sella, anterior hypopituitarism, pituitary

## Abstract

**Background/Objective:**

Primary empty sella (ES) is typically considered a benign incidental radiologic finding characterized by herniation of the subarachnoid space into the sella turcica and flattening of the pituitary gland. Emerging evidence suggests ES can be associated with varying degrees of clinically significant pituitary dysfunction, which is often underrecognized. We present a case highlighting the endocrine implications of ES and the need for comprehensive evaluation.

**Case Report:**

A 53-year-old woman was referred for abnormal thyroid function tests found on routine screening. She reported fatigue, cold intolerance, and cognitive slowing. Laboratory evaluation revealed central hypothyroidism, and a subsequent evaluation of anterior pituitary function revealed central adrenal insufficiency, hypogonadotropic hypogonadism, and growth hormone deficiency. There was no evidence of arginine vasopressin deficiency. Magnetic resonance imaging showed a primary ES with no visualized pituitary gland. Initiation of hormone replacement therapy subsequently resulted in significant clinical improvement.

**Discussion:**

This case illustrates that ES may represent a radiologic marker of evolving anterior hypopituitarism, rather than a benign variant. Without appropriate endocrine evaluation, such cases may be misdiagnosed or untreated, leading to worsened morbidity. Given the increasing use of advanced neuroimaging, incidental detection of ES is rising, making awareness of its potential endocrine consequences timely and relevant. Clinical vigilance and comprehensive endocrine assessment are essential to avoid delayed diagnosis and complications.

**Conclusion:**

Primary ES may herald progressive pituitary dysfunction. Clinicians should maintain a high index of suspicion and evaluate for central hormonal deficits when primary ES is identified incidentally.

**Highlights**

• Primary empty sella can present with evolving anterior hypopituitarism over time
• Comprehensive pituitary function testing is essential in incidental empty sella findings
• Early hormone replacement improves clinical and biochemical outcomes

**Clinical Relevance**

This case highlights the potential for significant endocrine dysfunction in primary empty sella, emphasizing the need for proactive hormonal evaluation even when initial imaging appears benign.

## Introduction

Primary empty sella (ES) is a radiological finding that refers to herniation of the subarachnoid space into the sella turcica resulting in flattening of the pituitary gland, often due to incompetence of the diaphragma sellae and frequently associated with increased intracranial pressure (ICP) in the absence of preceding pituitary pathology. On the other hand, secondary ES occurs following cranial surgery, irradiation, or insults such as ischemic damage to the hypothalamic-pituitary axis as a sequelae of stroke and Sheehan’s syndrome.^1^^,^^2^

With increased utilization of advanced neuroimaging, the incidental detection of ES has become more common, with prevalence estimates ranging from 2% to 35% in the general population.^3^^,^^4^ While ES has been traditionally considered as a benign, incidental radiologic finding, emerging literature suggests that a significant proportion of affected individuals may harbor varying degrees of clinically significant hypopituitarism, with reported rates ranging from 28% to 59%, with growth hormone, gonadotropin, and thyrotropin deficiencies the most common.^5^, ^6^, ^7^ Some patients present with multiple deficiencies or evolve into full panhypopituitarism. Hyperprolactinemia has also been observed in a subset of patients, attributed to disruption of dopaminergic inhibition.

Case reports have demonstrated acute presentations of ES with severe endocrine dysfunction congruent with adrenal insufficiency, severe hypothyroidism, or even arginine vasopressin deficiency, with symptoms including refractory hypoglycemia, hypotension, seizures, altered mental status, and hyponatremia, emphasizing the potential for the radiologic finding of ES to mask endocrine dysfunction until acute decompensation occurs.^8^, ^9^, ^10^, ^11^

We present a case of anterior hypopituitarism diagnosed following investigation for incidental abnormal thyroid function tests in a patient later found to have primary ES. The case emphasizes the importance of clinical vigilance and illustrates that ES, regardless of whether incidentally found or not, warrants comprehensive evaluation of pituitary function.

## Case Report

This is a case of a 53-year-old hyposthenic Caucasian female who was initially referred to the endocrinology clinic for abnormal thyroid function tests incidentally noted on routine laboratory work as part of a wellness exam. Other comorbidities include severe rheumatic mitral stenosis, heart failure with reduced ejection fraction, chronic obstructive pulmonary disease, and osteoarthritis, with no prior history of thyroid or pituitary disorders. She is gravida 3 para 1 (1201) and underwent a hysterectomy in her 30s. She was a nonsmoker and nonalcoholic drinker.

Thyroid testing dating back to 2022 showed persistently low free T4 with inappropriately normal thyroid-stimulating hormone values (Table). Total T3 (0.90; normal range: 0.60-1.80 ng/mL) was normal, and thyroid peroxidase antibodies were negative. The patient was not taking biotin or other agents that could cause thyroid assay interference. Symptoms included chronic fatigue, cognitive slowing, cold intolerance, and constipation. On examination, she appeared pale with blood pressure readings ranging from 90/50 to 100/70 mmHg but had no orthostatic changes or signs of fluid loss.

**Table:** Serial Thyroid Function Tests (2020–2023) and Anterior Pituitary Hormone Profile at Presentation (August 2023)

	2020	2022	4/2023	8/2023	Postreplacement
FT4 (0.60-1.60 ng/dL)	0.53	0.50	0.36	0.42	0.81
FT4 by ED (1.1-2.4 ng/dL)				0.5	
TSH (0.34-5.60 uIU/mL)	2.36	3.71	5.30	5.57	
Total T3 (0.60-1.8 ng/mL)				0.90	
8 am cortisol (>14 mcg/dL)				1.78	
Adrenocorticotropic hormone (pg/mL)				13	
Peak 60-min cortisol postcosyntropin stimulation (>14 mcg/dL)				4	
Estradiol (age-adjusted; <20-30 pg/mL)				<15	
Follicle-stimulating hormone (age-adjusted; 25-135 mIU/mL)				9	
Luteinizing hormone (age-adjusted; 14-52 mIU/mL)				2.5	
Insulin-like growth factor 1 (age-adjusted; 50-317 ng/mL)				21	
Prolactin (1.8-20.3 ng/mL)				2.84	

Abbreviations: *FT4* = free thyroxine; *TSH* = thyroid-stimulating hormone.

This table summarizes the patient's thyroid function tests—including FT4 and TSH—from 2020 through 2023, highlighting a progressive decline in FT4 levels with inappropriately normal to mildly elevated TSH, consistent with evolving central hypothyroidism. A comprehensive anterior pituitary hormone evaluation in August 2023 revealed multiple deficiencies, confirming anterior hypopituitarism. Results following hormone replacement are also included.

**Fig. 1:**
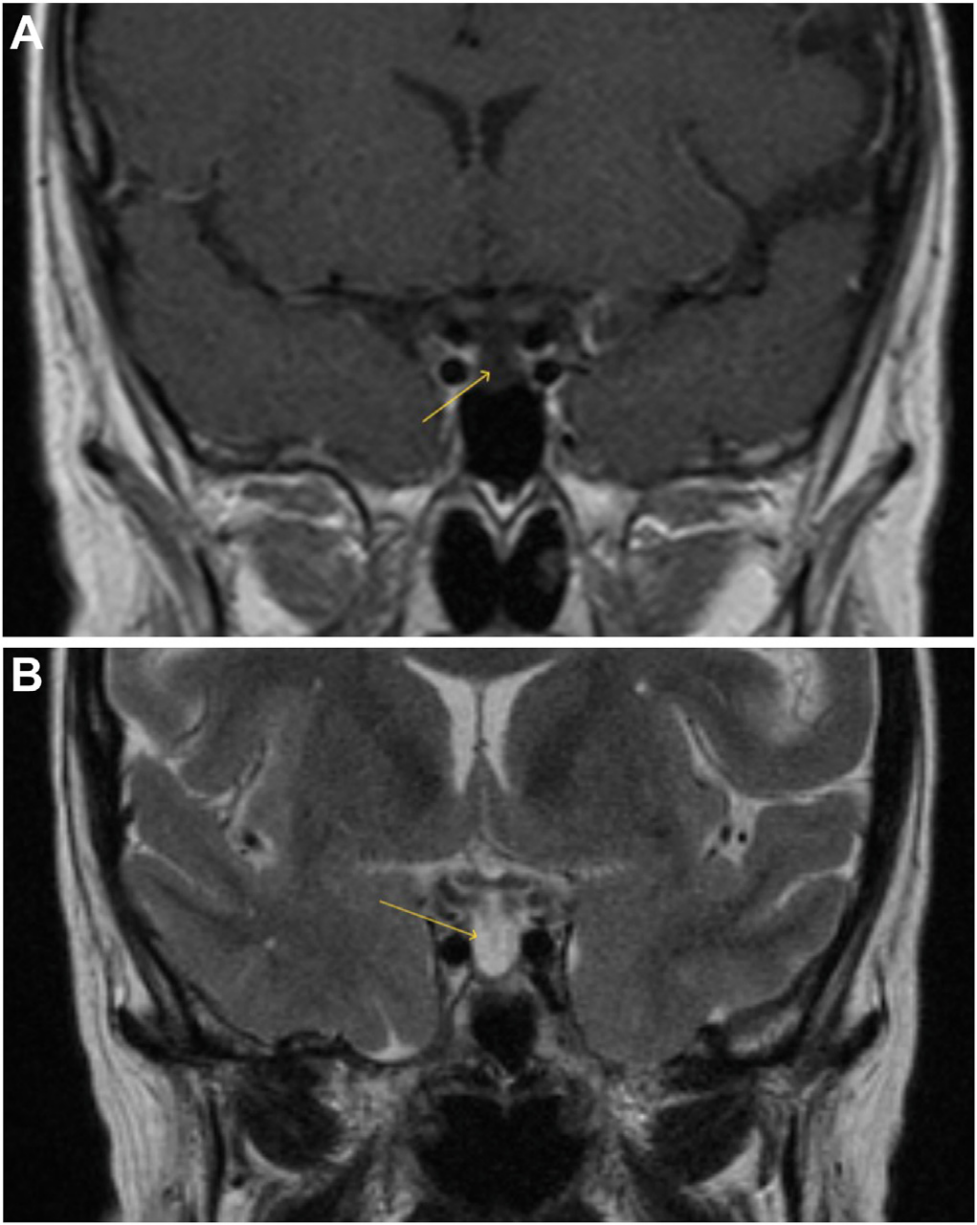
Coronal MRI of the pituitary gland showing empty sella. (A) T1-weighted image demonstrating herniation of the subarachnoid space into the sella turcica (yellow arrow) and a flattened pituitary gland. (B) T2-weighted image highlighting cerebrospinal fluid (CSF) signal within the sella with sellar ballooning (yellow arrow). *MRI* = magnetic resonance imaging.

**Fig. 2:**
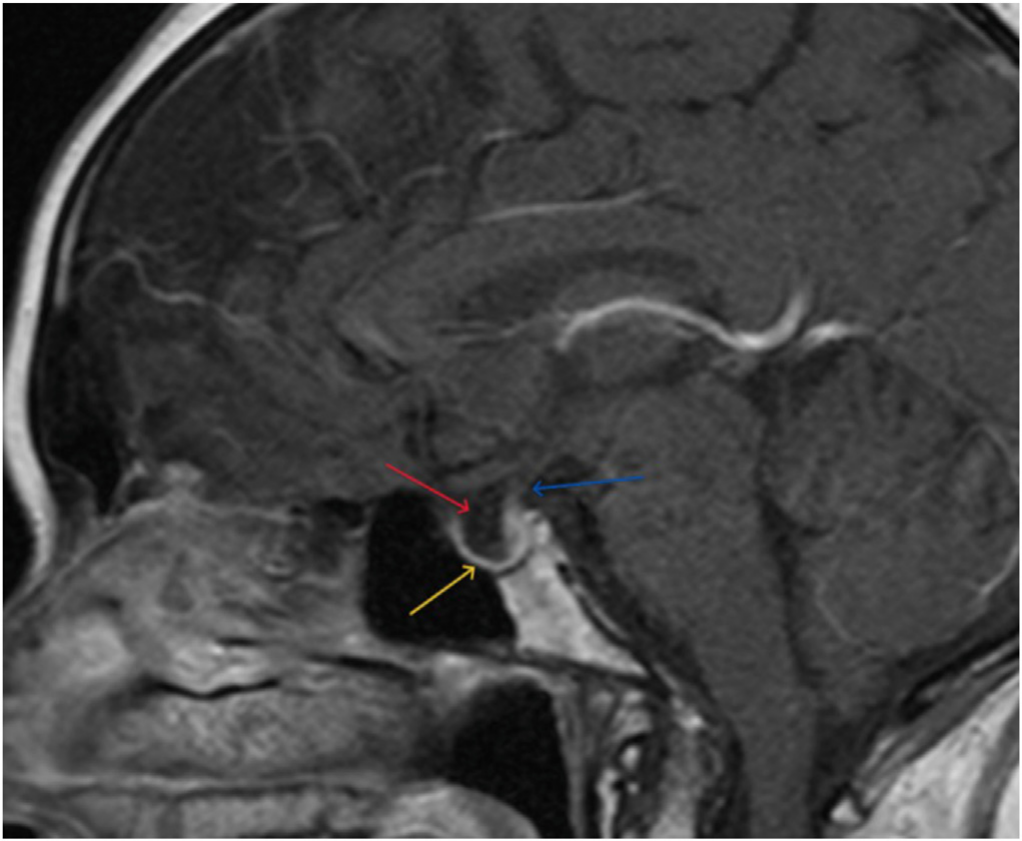
Sagittal T1-weighted postcontrast MRI of the pituitary gland demonstrating an empty sella with sellar ballooning. The sella turcica is enlarged and filled with cerebrospinal fluid (red arrow), causing marked flattening and thinning (yellow arrow) of the pituitary gland (measuring approximately 1 mm) along the sellar floor. The infundibulum (blue arrow) is noted to be stretched superiorly and deviated posteriorly. *MRI* = magnetic resonance imaging.

**Fig. 3:**
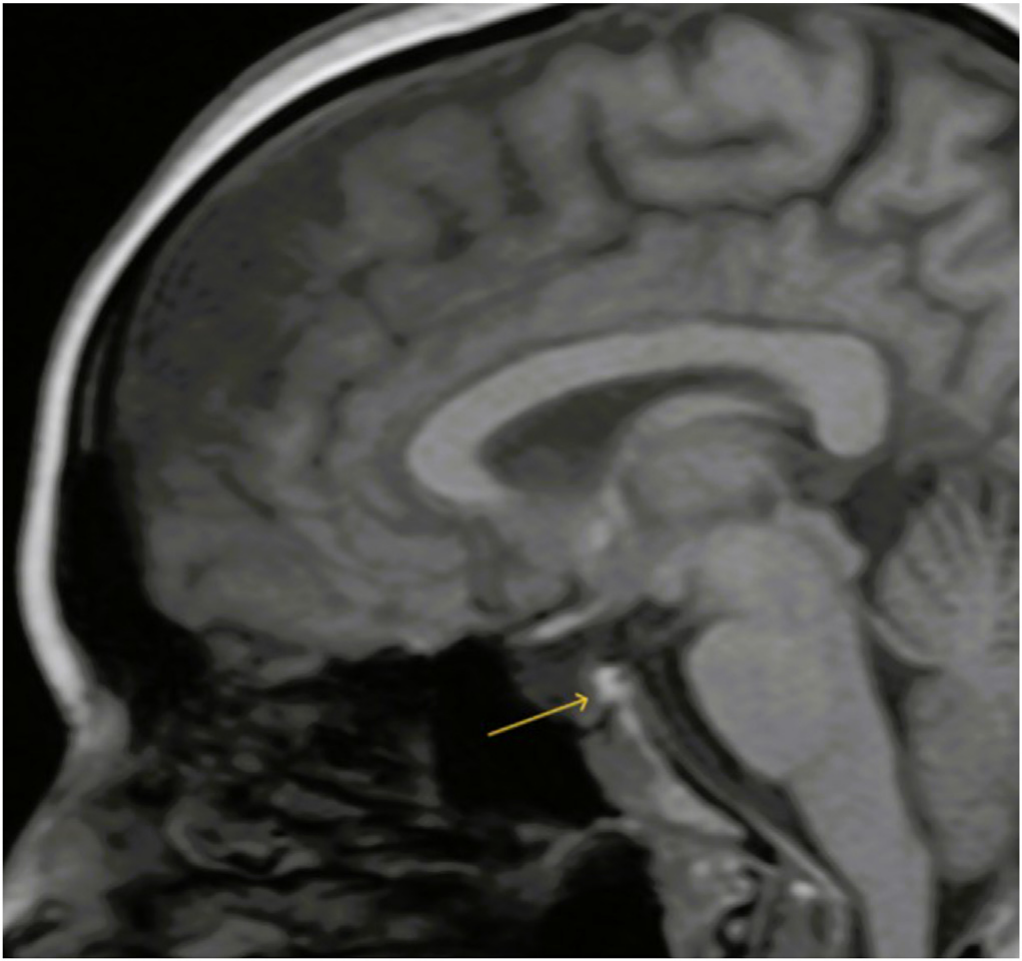
Sagittal T1-weighted MRI of the pituitary gland showing a characteristic hyperintense signal in the posterior pituitary (yellow arrow), indicative of preserved arginine vasopressin function. *MRI* = magnetic resonance imaging.

Free T4 by equilibrium dialysis (0.5; normal range: 1.1-2.4 ng/dL) confirmed central hypothyroidism. As warranted by the clinical picture, an 8 am fasting serum cortisol (1.78; normal range: >14 mcg/dL) and adrenocorticotropic hormone (ACTH) (13 pg/mL) levels were drawn and showed evidence of central adrenal insufficiency, which was confirmed by suboptimal response to cosyntropin stimulation (60-minute cortisol 4 mcg/dL; expected value >14 mcg/dL). Further workup revealed hypogonadotropic hypogonadism (estradiol <15 pg/mL, follicule-stimulating hormone 9 mIU/mL, luteinizing hormone 2.5 mIU/mL) and growth hormone deficiency (insulin-like growth factor-1 21; normal range: 50-317 ng/mL), leading to a diagnosis of anterior hypopituitarism. Prolactin (2.84; normal range: 1.8-20.3 ng/mL) and serum sodium were within normal limits, and there was no clinical suspicion for posterior pituitary involvement. Pituitary magnetic resonance imaging (MRI) revealed a flattened pituitary gland with cerebrospinal fluid (CSF) filling the sella turcica, consistent with primary ES (Figs. 1 and 2), and a hyperintense posterior pituitary signal on T1-weighted imaging (Fig. 3), indicating preserved arginine vasopressin function.

The patient was started on hydrocortisone replacement calculated per body surface area—10 mg in the morning and 10 mg in the late afternoon. Two days after hydrocortisone was started, weight-based levothyroxine replacement was introduced with marked clinical and symptom improvement at her 3-month follow-up visit. She reported improved energy, less daytime somnolence, normalization of blood pressure, and healthy weight gain, as well as resolution of pallor and cognitive fog. Growth hormone replacement was deferred due to the risk of fluid overload and edema in the context of systolic heart failure secondary to severe rheumatic mitral stenosis, for which valvular surgery has been planned.

## Discussion

This case illustrates the evolving understanding of primary ES as a potential marker for significant endocrine dysfunction. While traditionally considered a benign radiological entity, several large cohort and imaging studies show that up to 59% of individuals with primary ES demonstrate some degree of hypopituitarism, particularly involving the gonadotroph, somatotroph, and thyrotroph axes.^3^^,^^5^^,^^6^ Our patient initially presented with biochemical evidence of central hypothyroidism and was subsequently diagnosed with anterior hypopituitarism in the setting of an otherwise incidental radiologic finding of ES.

Studies have shown that pituitary dysfunction occurs more commonly in males (64%); in females, it is noted to be more prevalent in middle-aged women (22%) with a history of multiple pregnancies.^6^ Of note, a study demonstrated that pituitary insufficiency was more common in those with a normal-sized or deep sella, while ballooned sellae were typically associated with normal function, whereas the type of herniation and sellar bone changes were not predictive of hormonal abnormalities.^12^

A 2024 comprehensive review emphasized that although the majority of ES cases are benign and incidentally found, clinicians should maintain a high index of suspicion for hypopituitarism, particularly in the presence of nonspecific systemic symptoms or unexplained laboratory abnormalities such as hyponatremia, hypoglycemia, or hypotension.^12^^,^^13^ Cases of acute decompensation, including seizures and coma, have been reported as initial presentations of severe endocrine dysfunction in ES.^8^, ^9^, ^10^ Furthermore, literature highlights the diagnostic challenges associated with even partial ES and masked arginine vasopressin deficiency (formerly referred to as central diabetes insipidus), such as that seen in patients presenting with hyponatremia who were initially diagnosed with panhypopituitarism and subsequently developed overt polyuria and polydipsia after glucocorticoid therapy unmasked underlying vasopressin deficiency.^14^^,^^15^ These observations underscore the importance of clinical vigilance and comprehensive evaluation when pituitary dysfunction is suspected, as these can be masked and unmasked depending on dynamic physiologic stress or therapeutic interventions.^14^ Routine hormonal screening and appropriate MRI imaging can assist in early detection of endocrine dysfunction in cases of incidentally detected ES and vice versa.

Although the exact pathophysiology remains incompletely understood, proposed mechanisms for endocrine dysfunction include hypothalamic dysregulation, autoimmunity, chronic arachnoiditis, and impaired CSF dynamics wherein increased CSF pressure may cause compression of the pituitary gland or stalk, impairing hypothalamic-pituitary signaling.^7^ In our patient, there was no structural abnormality of the pituitary gland apart from the ES visualized on MRI, reinforcing the role of primary ES as a potential functional marker of underlying pituitary insufficiency. Some evidence suggests that ES itself may be the result of long-standing hypothalamic dysfunction rather than pituitary pathology per se^8^ as demonstrated by a blunted ACTH-cortisol response to hypoglycemia in the background of an exaggerated ACTH response to corticotropin-releasing hormone administration as well as a delayed thyroid-stimulating hormone response to thyrotropin-releasing hormone stimulation.^8^ An autoimmune process as a plausible mechanism has been proposed, as seen in a case of a 66-year-old woman with rheumatoid arthritis and unexplained hyponatremia and hypoglycemia, where MRI confirmed the diagnosis of ES syndrome and subsequent endocrine evaluation revealed panhypopituitarism.^16^ The presence of pituitary and cerebral antibodies in some cases suggests a potential mechanism underlying hypothalamic dysfunction, likely representing a distinct pathological process causing panhypopituitarism rather than a direct consequence of ES syndrome.^16^^,^^17^ Interestingly, certain patterns of sellar remodeling, such as a ballooned sella, have not been found to predict endocrine dysfunction.^12^

In addition to anterior pituitary dysfunction, ES can also be associated with idiopathic intracranial hypertension (IIH) and was found to be present in up to 94% of patients with IIH.^18^, ^19^, ^20^ Clinical signs of elevated ICP, such as headache, papilledema, and visual disturbances, along with perioptic nerve sheath distension on imaging, warrant further diagnostic evaluation, including cranial MRI with venography and lumbar puncture, to confirm IIH.^20^, ^21^, ^22^ Management of IIH focuses on lowering ICP primarily through weight loss, acetazolamide, and symptomatic treatment, with CSF shunting reserved for progressive vision loss.^21^^,^^22^ Neuro-ophthalmologic monitoring with visual acuity and perimetry is recommended at IIH diagnosis and during follow-up to prevent permanent optic nerve damage.^22^^,^^23^ Prognosis depends on timely ICP control, as untreated papilledema risks irreversible vision loss.^18^^,^^22^

This case highlights the need for a multidisciplinary approach and heightened awareness of ES and the associated endocrine dysfunction as opposed to the traditionally held notion of ES being a clinically insignificant radiologic finding and as a potentially underrecognized cause of acute and chronic pituitary insufficiency. Pituitary function in ES may fluctuate over time,^4^^,^^12^ necessitating long-term endocrine follow-up as well.

## Conclusion

Primary ES may reflect underlying hypothalamic-pituitary dysfunction rather than a benign imaging finding. Routine endocrine screening should be considered in patients with ES and unexplained systemic symptoms. Early diagnosis and hormone replacement can lead to significant improvement in quality of life and prevent complications.

## Declaration of Generative AI and AI-Assisted Technologies in the Writing Process

During the preparation of this work, the author used a large language model (ChatGPT-4) to assist with proofreading and language refinement. After using this tool/service, the author reviewed and edited the content as needed and takes full responsibility for the integrity and accuracy of the final manuscript.

## Patient Consent

Informed consent was obtained from the patient for publication of this case report. Efforts have been made to ensure anonymity and protect patient identity.

## Disclosure

The author has no conflicts of interest to disclose.
